# Supplementary material for: A modified modeling and dynamical behavior analysis method for fractional-order positive Luo converter
Source: PLoS One. 2020 Aug 14;15(8):e0237169. doi: 10.1371/journal.pone.0237169 (PMC7428135; doi:10.1371/journal.pone.0237169)
Supplement: S1 Appendix — The solutions of x0, x1, x2 in Eqs (Eq (20a))–(20c) can be obtained by the derivation. (PDF) [file pone.0237169.s001.pdf]

S1 Appendix

The solutions of $\mathbf{x}_0$, $\mathbf{x}_1$, $\mathbf{x}_2$ in Eqs (20a)-(20c) could be obtained by the following derivation. Using Eqs (11a) and (15a), we get that

$$\mathbf{f}_0 = s_0\mathbf{x}_0 = b_0\mathbf{a_{00}} + b_1\mathbf{a_{00}}e^{j\tau} + \bar{b}_1\mathbf{a_{00}}e^{-j\tau} = \mathbf{f}_{0k} + \mathbf{R}_1 \tag{S1.1}$$

As $\mathbf{f}_{0k}$ includes the same harmonics as the main wave $\mathbf{x}_0$, we could deduce

$$\mathbf{f}_{0k} = b_0\mathbf{a_{00}} \tag{S1.2}$$

and the rest harmonics of $\mathbf{f}_0$ stands for $\mathbf{R}_1$.

Substituting $\mathbf{f}_{0k}$ and $\mathbf{x}_0$ into (20a), and using the DC components only on the right side, we obtain

$$\mathbf{G}_{10}\mathbf{a}_{00} + \mathbf{G}_2 b_0\mathbf{a}_{00} = \mathbf{H_1} + b_0\mathbf{H}_2 \tag{S1.3}$$

where the matrix $\mathbf{G}_{10} = \mathbf{G}_1(0^\alpha, 0^\beta, 0^\gamma)$. Eq (S1.2) could be overwritten into the following form

$$\left(\begin{bmatrix} \frac{R_{in}}{L} & \frac{1}{L} & -\frac{1}{L} \\ -\frac{1}{C_o} & \frac{1}{R_oC_o} & 0 \\ \frac{1}{C_b} & 0 & 0 \end{bmatrix} + D\begin{bmatrix} -\frac{R_{in}}{L} & -\frac{1}{L} & 0 \\ \frac{1}{C_o} & 0 & 0 \\ 0 & 0 & \frac{1}{R_{in}C_b} \end{bmatrix}\right)\begin{bmatrix} I_{00} \\ V_{o00} \\ V_{b00} \end{bmatrix} = \begin{bmatrix} \frac{(1-D)V_{in}}{L} \\ 0 \\ \frac{V_{in}}{R_{in}C_b} \end{bmatrix} \tag{S1.4}$$

Hence, $\mathbf{x}_0 = \mathbf{a}_{00}$ is obtained, and we should go further to get $\mathbf{x}_1$. Firstly, we have to find the spectral content of $\mathbf{x}_1$. From Eqs (S1.1) and (S1.2), it could be obtained that

$$\mathbf{R}_1 = b_1\mathbf{a_{00}}e^{j\tau} + \bar{b}_1\mathbf{a_{00}}e^{-j\tau} \tag{S1.5}$$

As it could be seen in (20b), the spectral content of $\mathbf{R}_1$ depends on the spectral content of $\mathbf{x}_1$. Then the spectral content set of $\mathbf{x}_1$ is $\{\boldsymbol{E}_1\} = \{1\}$. We have to assume that $\mathbf{x}_1$ could be

$$\mathbf{x}_1 = \mathbf{a}_{11}e^{j\tau} + \bar{\mathbf{a}}_{11}e^{-j\tau} \tag{S1.6}$$

where $\mathbf{a}_{11} = [I_{11}\ V_{o11}\ V_{b11}]^T$. Introducing s_0, s_1, $\mathbf{x}_0$ and $\mathbf{x}_1$ into $\mathbf{f}_1$, the following expression could be concluded

$$\mathbf{f}_1 = s_0\mathbf{x}_1 + s_1\mathbf{x}_0 = \mathbf{f}_{1k} + \mathbf{R}_2 = b_0\mathbf{a}_{11}e^{j\tau} + b_0\bar{\mathbf{a}}_{11}e^{-j\tau} + (b_1\bar{\mathbf{a}}_{11} + \bar{b}_1\mathbf{a}_{11}) + (b_1\mathbf{a}_{11} + b_2\mathbf{a}_{00})e^{j2\tau} + b_3\mathbf{a}_{00}e^{j3\tau} + c.c \tag{S1.7}$$

in which $c.c$ denotes the complex conjugate items. The above equation contains the items with the exponents $e^{-j2\tau}$ and $e^{-j3\tau}$.

Considering the component $\mathbf{f}_{1k}$ should consist of all the items that have the same harmonics as $\mathbf{x}_1$ in $\mathbf{f}_1$, it could be represented as

$$\mathbf{f}_{1k} = b_0\mathbf{a}_{11}e^{j\tau} + b_0\bar{\mathbf{a}}_{11}e^{-j\tau} \tag{S1.8}$$

Thus, we can solve Eq (20b) by substituting $\mathbf{f}_{1k}$ and $\mathbf{R}_1$. If the right side is adjusted by considering $\{\boldsymbol{E}_1\}$, Eq (20b) could be rewritten as

$$\mathbf{G}_1(p^\alpha, p^\beta, p^\gamma)\mathbf{a}_{11}e^{j\tau} + \mathbf{G}_2(b_0\mathbf{a}_{11}e^{j\tau} + b_1\mathbf{a_{00}}e^{j\tau}) = b_1\mathbf{H}_2e^{j\tau} \tag{S1.9}$$

According to Eq (1), there are $p^\alpha e^{j\omega t} = (j\omega)^\alpha e^{j\omega t}$ and $p^\beta e^{j\omega t} = (j\omega)^\beta e^{j\omega t}$. Moreover, $\mathbf{a}_{11}$ could be solved by

$$(\mathbf{G}_{11} + \mathbf{G}_2 b_0)\mathbf{a}_{11} = b_1\mathbf{H}_2 - \mathbf{G}_2 b_1\mathbf{a_{00}} \tag{S1.10}$$

Here, the coefficient $\mathbf{G}_{11}$ = $\mathbf{G}_1$ (($j\omega$)$^\alpha$, ($j\omega$)$^\beta$, ($j\omega$)$^\gamma$). Therefore, Eq (S1.3) can be overwritten into

$$\left(\begin{bmatrix} (j\omega)^{\alpha}+\frac{R_{in}}{L} & \frac{1}{L} & -\frac{1}{L} \\ -\frac{1}{C_o} & (j\omega)^{\beta}+\frac{1}{R_oC_o} & 0 \\ \frac{1}{C_b} & 0 & (j\omega)^{\gamma} \end{bmatrix}+D\begin{bmatrix} -\frac{R_{in}}{L} & -\frac{1}{L} & 0 \\ \frac{1}{C_o} & 0 & 0 \\ 0 & 0 & \frac{1}{R_{in}C_b} \end{bmatrix}\right)\begin{bmatrix} I_{11} \\ V_{o11} \\ V_{b11} \end{bmatrix}$$
$$=\begin{bmatrix} \frac{-b_1V_{in}}{L} \\ 0 \\ \frac{b_1V_{in}}{R_{in}C_b} \end{bmatrix}-\begin{bmatrix} -\frac{R_{in}}{L} & -\frac{1}{L} & 0 \\ \frac{1}{C_o} & 0 & 0 \\ 0 & 0 & \frac{1}{R_{in}C_b} \end{bmatrix}\begin{bmatrix} b_1I_{00} \\ b_1V_{o00} \\ b_1V_{b00} \end{bmatrix} \tag{S1.11}$$

Hence, both $\mathbf{a}_{11}$ and $\mathbf{x}_{11}$ are obtained. Because of the influence of ($j\omega$)$^\alpha$, ($j\omega$)$^\beta$ and ($j\omega$)$^\gamma$, the relationship between the harmonic magnitude of $\mathbf{x}_1$ and the orders of α, β and γ is non-linear. In order to find the spectral content of $\mathbf{x}_1$, the spectral contents of $\mathbf{R}_2$ should be considered. From Eqs (S1.7) and (S1.8), $\mathbf{R}_2$ could be found that

$$\mathbf{R}_2=(b_1\bar{\mathbf{a}}_{11}+\bar{b}_1\mathbf{a}_{11})+(b_1\mathbf{a}_{11}+b_2\mathbf{a}_{00})e^{j2\tau}+b_3\mathbf{a}_{00}e^{j3\tau}+c.c \tag{S1.12}$$

Based on the above equation and Eq (20c), the spectral content set of $\mathbf{x}_2$ is $\{E_2\}$ = {0, 2, 3}.Thus, $\mathbf{x}_2$ can be expressed as

$$\mathbf{x}_2=\mathbf{a}_{02}+\mathbf{a}_{22}e^{j2\tau}+\bar{\mathbf{a}}_{22}e^{-j2\tau}+\mathbf{a}_{32}e^{j3\tau}+\bar{\mathbf{a}}_{32}e^{-j3\tau} \tag{S1.13}$$

where $\mathbf{a}_{02}$ = $[I_{02}\ V_{o02}\ V_{b02}]^T$ gives the corrections of DC components in $\mathbf{a}_{00}$. Here, $\mathbf{a}_{22}$ = $[I_{22}\ V_{o22}\ V_{b22}]^T$ is the second harmonic magnitude of $\mathbf{x}_2$, while $\mathbf{a}_{22}$ = $[I_{32}\ V_{o32}\ V_{b32}]^T$ is the third harmonic magnitude. By substituting s_i and $\mathbf{x}_i$ (i = 0, 1, 2) into $\mathbf{f}_2$, considering the component $\mathbf{f}_{2k}$ should consist of the same harmonics as $\mathbf{x}_2$, the following expressions of $\mathbf{f}_{2k}$ and $\mathbf{R}_3$ could be deduced

$$\mathbf{f}_{2k}=b_0\mathbf{a}_{02}+(b_0\mathbf{a}_{22}+\bar{b}_1\mathbf{a}_{32}+b_3\bar{\mathbf{a}}_{11})e^{j2\tau}+(b_0\mathbf{a}_{32}+b_1\mathbf{a}_{22}+b_2\mathbf{a}_{11})e^{j3\tau}+c.c \tag{S1.14a}$$

$$\mathbf{R}_3=(b_1\mathbf{a}_{02}+\bar{b}_1\mathbf{a}_{22}+b_2\bar{\mathbf{a}}_{11})e^{j\tau}+(b_1\mathbf{a}_{32}+b_3\mathbf{a}_{11}+b_4\mathbf{a}_{00})e^{j4\tau}+b_5\mathbf{a}_{00}e^{j5\tau}+c.c \tag{S1.14b}$$

Introducing $\mathbf{x}_1$, $\mathbf{f}_{2k}$ and $\mathbf{R}_2$, utilizing the harmonic balance method, we obtain the following three equations:

$$(\mathbf{G}_{10}+\mathbf{G}_2b_0)\mathbf{a}_{02}=-\mathbf{G}_2(b_1\bar{\mathbf{a}}_{11}+\bar{b}_1\mathbf{a}_{11}) \tag{S1.15a}$$

$$(\mathbf{G}_{12}+\mathbf{G}_2b_0)\mathbf{a}_{22}=b_2\mathbf{H}_2-\mathbf{G}_2(b_3\bar{\mathbf{a}}_{11}+b_2\mathbf{a}_{00}+b_1\mathbf{a}_{11}) \tag{S1.15b}$$

$$(\mathbf{G}_{13}+\mathbf{G}_2b_0)\mathbf{a}_{32}=b_3\mathbf{H}_2-\mathbf{G}_2(b_1\mathbf{a}_{22}+b_2\mathbf{a}_{11}+b_3\mathbf{a}_{00}) \tag{S1.15c}$$

Similarly, $\mathbf{G}_{1m}$ = $\mathbf{G}_1$ (($jm\omega$)$^\alpha$, ($jm\omega$)$^\beta$, ($jm\omega$)$^\gamma$) (m = 0, 2, 3), Eq (S1.15) could be overwritten as

$$\left(\begin{bmatrix} \frac{R_{in}}{L} & \frac{1}{L} & -\frac{1}{L} \\ -\frac{1}{C_o} & \frac{1}{R_oC_o} & 0 \\ \frac{1}{C_b} & 0 & 0 \end{bmatrix} + D\begin{bmatrix} -\frac{R_{in}}{L} & -\frac{1}{L} & 0 \\ \frac{1}{C_o} & 0 & 0 \\ 0 & 0 & \frac{1}{R_{in}C_b} \end{bmatrix}\right)\begin{bmatrix} I_{02} \\ V_{o02} \\ V_{b02} \end{bmatrix}$$

$$= -\begin{bmatrix} -\frac{R_{in}}{L} & -\frac{1}{L} & 0 \\ \frac{1}{C_o} & 0 & 0 \\ 0 & 0 & \frac{1}{R_{in}C_b} \end{bmatrix}\begin{bmatrix} \bar{b}_1 I_{11} + b_1 \bar{I}_{11} \\ \bar{b}_1 V_{o11} + b_1 \bar{V}_{o11} \\ \bar{b}_1 V_{b11} + b_1 \bar{V}_{b11} \end{bmatrix} \tag{S1.16a}$$

$$\left(\begin{bmatrix} (j2\omega)^{\alpha} + \frac{R_{in}}{L} & \frac{1}{L} & -\frac{1}{L} \\ -\frac{1}{C_o} & (j2\omega)^{\beta} + \frac{1}{R_oC_o} & 0 \\ \frac{1}{C_b} & 0 & (j2\omega)^{\gamma} \end{bmatrix} + D\begin{bmatrix} -\frac{R_{in}}{L} & -\frac{1}{L} & 0 \\ \frac{1}{C_o} & 0 & 0 \\ 0 & 0 & \frac{1}{R_{in}C_b} \end{bmatrix}\right)\begin{bmatrix} I_{22} \\ V_{o22} \\ V_{b22} \end{bmatrix}$$

$$== \begin{bmatrix} \frac{-b_2 V_{in}}{L} \\ 0 \\ \frac{b_2 V_{in}}{R_{in}C_b} \end{bmatrix} - \begin{bmatrix} -\frac{R_{in}}{L} & -\frac{1}{L} & 0 \\ \frac{1}{C_o} & 0 & 0 \\ 0 & 0 & \frac{1}{R_{in}C_b} \end{bmatrix}\begin{bmatrix} b_1 I_{11} + b_2 I_{00} + b_3 \bar{I}_{11} \\ b_1 V_{o11} + b_2 V_{o00} + b_3 \bar{V}_{o11} \\ b_1 V_{b11} + b_2 V_{b00} + b_3 \bar{V}_{b11} \end{bmatrix} \tag{S1.16b}$$

$$\left(\begin{bmatrix} (j3\omega)^{\alpha} + \frac{R_{in}}{L} & \frac{1}{L} & -\frac{1}{L} \\ -\frac{1}{C_o} & (j3\omega)^{\beta} + \frac{1}{R_oC_o} & 0 \\ \frac{1}{C_b} & 0 & (j3\omega)^{\gamma} \end{bmatrix} + D\begin{bmatrix} -\frac{R_{in}}{L} & -\frac{1}{L} & 0 \\ \frac{1}{C_o} & 0 & 0 \\ 0 & 0 & \frac{1}{R_{in}C_b} \end{bmatrix}\right)\begin{bmatrix} I_{32} \\ V_{o32} \\ V_{b32} \end{bmatrix} \tag{S1.16c}$$

$$== \begin{bmatrix} \frac{-b_3 V_{in}}{L} \\ 0 \\ \frac{b_3 V_{in}}{R_{in}C_b} \end{bmatrix} - \begin{bmatrix} -\frac{R_{in}}{L} & -\frac{1}{L} & 0 \\ \frac{1}{C_o} & 0 & 0 \\ 0 & 0 & \frac{1}{R_{in}C_b} \end{bmatrix}\begin{bmatrix} b_1 I_{22} + b_2 I_{11} + b_3 I_{00} \\ b_1 V_{o22} + b_2 V_{o11} + b_3 V_{o00} \\ b_1 V_{b22} + b_2 V_{b11} + b_3 V_{b00} \end{bmatrix}$$

In this way, $\mathbf{a}_{m2} = [I_{m2}\ V_{om2}\ V_{bm2}]^T$ (m = 0,2,3) could be obtained, and the correction $\mathbf{x}_2$ could be obtained.
