# Supplementary material for: A modified modeling and dynamical behavior analysis method for fractional-order positive Luo converter
Source: PLoS One. 2020 Aug 14;15(8):e0237169. doi: 10.1371/journal.pone.0237169 (PMC7428135; doi:10.1371/journal.pone.0237169)
Supplement: S2 Appendix — The parameters in the approximation circuit of the fractional-order components are deduced. (PDF) [file pone.0237169.s002.pdf]

S2 Appendix

The Approximation circuits of the fractional-order components are deduced by the following derivation.

Considering the frequency domain features, the impedance Z_L of the fractional inductor with an order of λ, and the impedance of Z_C of the fractional capacitor with an order of μ could be respectively defined as the following forms

$$Z_L = (j\omega)^{\lambda} L = (\omega)^{\lambda} L[\cos(\frac{\lambda\pi}{2}) + j\sin(\frac{\lambda\pi}{2})] \quad \text{(S2.1a)}$$

$$Z_C = \frac{1}{(j\omega)^{\mu} C} = \frac{1}{(\omega)^{\mu} C}[\cos(\frac{\mu\pi}{2}) - j\sin(\frac{\mu\pi}{2})] \quad \text{(S2.1b)}$$

Here, the frequency-domain properties of Z_L and Z_C are as follows:

Magnitude: Z_L has a slope of 20λ dB/dec, and Z_C has a slope of -20μ dB/dec.

Phase: the angle value of Z_L is $\lambda\pi/2$, while the angle value of Z_C is $-\mu\pi/2$.

Using the method of the undetermined coefficients, we could get the impedances of the approximate circuit models:

$$\frac{1}{L}s^{-\alpha} = \sum_{i=1}^{n} \frac{1/L_{Li}}{s + R_{Li}/L_{Li}} + \frac{1}{R_{Lin}} \quad \text{(S2.2a)}$$

$$\frac{1}{C}s^{-\beta} = \sum_{i=1}^{n} \frac{1/C_{Ci}}{s + 1/R_{Ci}C_{Ci}} + R_{Cin} \quad \text{(S2.2b)}$$

where all the parameters in the above equations could be calculated by the comprehensive approach of the impedance networks.

For the fractional inductors and capacitors, the maximum discrepancy between the amplitude-frequency response of the approximate circuit impedance and that of the theoretical impedance is selected to be less 2 dB. Then the computational values of R_{Li} and L_{Li} for the inductor, with the order α = 0.8 and α = 0.9, are determined as listed in Table S2.1. Considering that some resistors in Table S2.1 have extremely small values, cement resistors with approximate values will be chosen in the actual experiments. Thus, the approximate actual values are also listed in Table S2.1, together with the values calculated by the synthesis method.

Table S2.1. The values of R_{Li} and L_{Li} for the fractional-order inductors with different α.

α	0.8				0.9			
i	$R_{Li}(\Omega)$		$L_{Li}(mH)$		$R_{Li}(\Omega)$		$L_{Li}(mH)$	
	Computational	Actual	Computational	Actual	Computational	Actual	Computational	Actual
in	0	0	\	\	0	0	\	\
1	5.5853*k*	5.6*k*	0.0418	0.042	394.75*k*	395*k*	0.2367	0.237
2	585.6896	590	0.0781	0.078	3.9635*k*	4.0*k*	0.3964	0.396
3	58.7139	59	0.1392	0.139	39.6360	40	0.6612	0.661
4	5.8722	6	0.2476	0.247	0.3964	400*m*	1.1029	1.1
5	0.5872	600*m*	0.4403	0.440	0.0039	4*m*	1.8332	1.8
6	0.0587	60*m*	0.7830	0.783	0.0160*m*	1*m*	1.2321	1.2
7	0.0059	6*m*	1.3902	1.4				
8	0.5695*m*	1*m*	2.4015	2.4				
9	0.2641*m*	1*m*	1.9804	2.0				

Moreover, the fractional-order capacitors can also be constructed by using the same way depicted in the above analysis. Thus, the values of R_{Ci} and C_{Ci} for capacitors (C_o =10 μF) with the order β = 0.8 and β = 0.9 are determined as in Table S2.2, where the actual values are included.

Table S2.2. The values of R_{Ci} and C_{Ci} for the fractional-order capacitors with different β.

β	0.8				0.9			
i	$R_{Ci}(\Omega)$		$C_{Ci}(\mu F)$		$R_{Ci}(\Omega)$		$C_{Ci}(\mu F)$	
	Computational	Actual	Computational	Actual	Computational	Actual	Computational	Actual
i	0	0	\	\	0	0	\	\
1	0.0179	20m	0.41885	0.4	0.25330m	1m	2.3667	2.4
2	0.1707	200m	0.78104	0.78	25.2m	30m	3.9639	4.0
3	1.7031	1.7	1.3924	1.4	2.5227	2.5	6.6123	6.6
4	17.0291	17	2.4763	2.5	252.2781	250	11.03	11
5	170.2903	170	4.4036	4.4	25.318k	25.3k	18.333	18
6	1.7031k	1.7k	7.8302	7.8	6.2834M	6.28M	12.322	12
7	17.058k	17.1k	13.902	14				
8	175.59k	175.6k	24.016	24				
9	3.7865M	3.8M	19.805	20				

Similarity, for the capacitor (C_b = 47 μF) with the order γ = 0.95, considering the number of the $R_{Ci}C_{Ci}$ blocks, the maximum discrepancy between the approximate lines and the desired curves of the frequency-domain properties is chosen to be 1 dB. Table S2.3 shows the computational and actual values of R_{Ci} and C_{Ci} for the capacitor with the order γ = 0.95.

Table S2.3. The values of R_{Ci} and C_{Ci} for the fractional-order capacitor with γ = 0.95.

γ	0.95			
i	$R_{Ci}(\Omega)$		$C_{Ci}(\mu F)$	
	Computational	Actual	Computational	Actual
in	0	0	\	\
1	0.036439m	1m	72.357	72
2	0.0036	3m	92.404	92
3	0.3636	400m	117.75	117
4	36.3614	36	150.04	150
5	3.6584k	3.66k	190.02	190
6	1.6864M	1.69	52.531	52

What's more, Fig S2.1 shows the bode diagrams of the approximate circuits for the fractional-order components, in which the black solid curves stand for the ideal result computed based on the Eqs (S2.2a) and (S2.2b). At the same time, the red dash lines represent the devices acquired by comprehensive approach, and the equivalent components approximated by actual devices are denoted by the blue dot plots. As seen in this figure, the magnitude lines are in good agreement with each other in the setting frequency range. In the phase curves, the fractional impedances of the approximation circuits fluctuate around the theoretical values within a certain frequency band. Reducing the maximum error of the amplitude response plot could improve the accuracy of the results. However, this could also greatly increase the number of the $R_{Ci}C_{Ci}$ or $R_{Li}L_{Li}$ blocks in each chain unit.

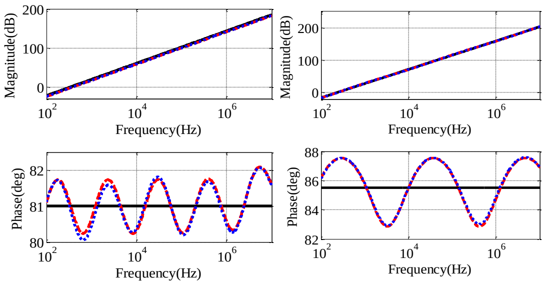

(a) (b)

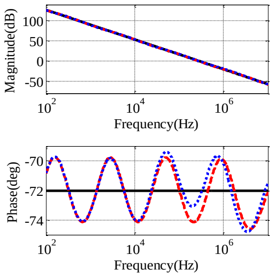

(c)

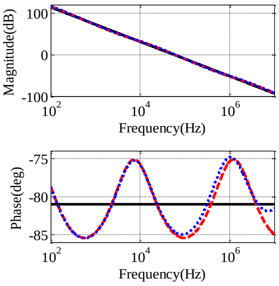

(d)

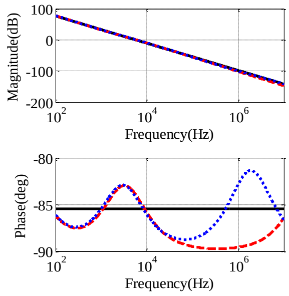

(e)

Fig S2.1. Bode diagrams of the constructed fractional components under different orders (a) the 0.8-order inductor L; (b) the 0.9-order inductor L; (c) the 0.8-order capacitor C_o; (d) the 0.9-order capacitor C_o; (e) the 0.95-order capacitor C_b.
