# Supplementary material for: A modified modeling and dynamical behavior analysis method for fractional-order positive Luo converter
Source: PLoS One. 2020 Aug 14;15(8):e0237169. doi: 10.1371/journal.pone.0237169 (PMC7428135; doi:10.1371/journal.pone.0237169)
Supplement: Attachment — Submitted filename: Reply to Editor.doc [file pone.0237169.s009.doc]

**Reply to Editor:**

Thank you very much for your valuable comments and good suggestions that greatly helped to improve our manuscript. At the same time, we should thank for your time and efforts.

We have carefully considered your valuable comments and good suggestions. In the following, we are going to explain how your comments have been taken into account in the revision.

**Response to comment 1:** Please explain the rationale for the development of your model in light of recent research in this area, clearly indicating which problem with existing models you are addressing.

**Response:** We are very sorry for not clearly indicating which problem with existing models we are addressing. Bases on your comment, we readjust the structure and make some modification in the introduction. The changes are shown in manuscript_ revision_v1.0.doc. We hope these modifications could help readers to understand this paper more deeply.

**Response to comment 2:** Please clearly report at the beginning of your methods or results section which were the key performance measures used to establish the validity and utility of your models. Please also report clearly which statistical analysis was used to establish robustness of performance measures.

**Response:** Terribly sorry for not reiterating the key performance measures used to establish the validity and utility of your models. We add these contents in the beginning of Section 3 and Section 5. The changes are shown in manuscript_ revision_v1.0.doc.

**Response to comment 3:** Whilst we note that the performance of your model has been compared to other methods, the manuscript is lacking a thorough discussion of the methods chosen for comparison and justification as to why they were chosen. Please modify your manuscript as necessary.

**Response:** We have added these comparisons and justifications in both Introduction and Section 5. The modifications are presented in manuscript_ revision_v1.0.doc.

**Response to comment 4:** As such we ask that you provide access to the Matlab code/scripts used in your study, either in the supplemental files or by providing a link to a database where they can be found.

**Response:** We have added these Matlab c-scripts of your study in the supporting information section.

**Response to comment 5:** We therefore request that you revise the text to fix the grammatical errors and improve the overall readability of the text before we send it for review.

**Response:** We have modified the expressions and done much work in language editing. We have the manuscript copyedited by

**Jingsu Yu**

*She is the post doctoral fellow, University of Toronto, Toronto, Canada. And she received the Ph.D. degrees in electrical engineering from the University of Toronto in 2019. Her research interests include integrated DC-DC converter and SoC design.*

**Shuai Shao**

*He is the [associate professor](http://www.youdao.com/w/associate professor/" \l "keyfrom=E2Ctranslation) of Zhejiang University, Hangzhou, China. And he received the Ph.D. degrees in electrical engineering from the University of Nottingham, Nottingham, UK, in 2016. His research interests include switching power supply design and microgrid converter control.*

**Dazheng Feng**

*He is the [professor](http://www.youdao.com/w/associate professor/" \l "keyfrom=E2Ctranslation) of Xidian University, Xi’an, China.*
